# Personal

**Published:** 1912-03

**Authors:** 


### PERSONAL

DR. M. A. KING of Rochester, has moved from 80 Clinton Avenue, South to 21 East Avenue. Office hours, 1 to 3 and 7 to 8.

DR. G. A. HITZEL of Buffalo, has recently been elected to a fifth term as President of the German-American Alliance.

DR. RALPH ROBINSON, Health Physician of Lackawanna, has recently recommended the appointment of a salaried nurse to look after the 1,300 school children of that city.

DR. W. A. MACPHERSON is agitating the establishment of a hospital for the Tonowandas, estimating that $75,000 is spent annually for the care of Tonawanda patients in Buffalo hospitals.

DR. WILLIAM B. BARTLETT of Castile is Captain of the Castile Sportsman's Club.

DR. F. E. FRONCZAK of Buffalo, delivered an address on tuberculosis at the East Side Business Men and Taxpayers' Association meeting, Feb. 6.

DR. LUDWIK SCHROETER of Buffalo, is going abroad for a number of months.

DR. ANNA LOUISE STRONG has recently visited Buffalo for the purpose of planning the Child Welfare Exhibit which will be held in Convention Hall, May 27—June 3.

DR. GEORGE A. STESEL of Buffalo, has resigned as probation officer of the juvenile court, on account of the recent ruling requiring the devotion of the entire time to the duties of the office —at a salary of $1,200. (This reminds us of the time when we resigned as city physician, after having earned the whole year's salary in a month and having made about 300 vaccinations for good measure. The Overseer of the Poor telephoned to see if anything he had done had given offense. He was one of the kindest-hearted men that ever lived, but he could not think of any other reason for the resignation.)

---

DR. BERNARD COHEN has removed from Niagara St., to 568 Lafayette Avenue, Buffalo.

---

DR. J. H. GLASS of Utica and Dr. G. Alden Blumer of Providence, R. I., sailed Feb. 3, for Panama and Central American ports.

---

DR. J. G. KILBOURNE of Utica sailed for Panama Feb. 21.

---

DRS. FREDERICK R. FORD and F. H. Peck of Utica sailed Feb. 24, for a six months' trip to Europe, their itinerary including Paris, Munich, Vienna, Dresden, Berlin, Rotterdam and London.

---

DR. F. H. PECK of Utica has recently been elected President of the Yahnundasis Golf Club.

---

THE editor acknowledges with thanks the hospitality of Dr. Charles W. Hennington and the courteous and patient attention of the Rochester Academy of Science, Feb. 12.

---

DR. JOHN M. SWAN, formerly of Philadelphia, and lately Superintendent of the Glens Springs Sanitarium, has re-entered private practice at 403 Park Ave., Rochester.

---

DR. C. C. SUTTER has also recently come to Rochester from Watkins and has located at 275 Alexander St.

---

DR. C. F. CHAFFE has been appointed County Bacteriologist of Monroe Co., the laboratory being located at 275 Alexander St, Rochester.

---

DR. CARL VON NOORDEN of Vienna has been engaged by the N. Y. Post-Graduate Medical School for a series of lectures on metabolism, to be delivered in October, 1912.

Dr. Dewitt G. Wilcox of Boston, formerly of Buffalo, has been appointed to the editorial staff of the New England Medical Gazette.

---

T'. Guilford Smith, LL.D. of Buffalo has completed his term of office as Regent of the University of the State of N. Y. and has been succeeded by Hon. Adelbert Moot of the same city. Hon. Lucius N. Littauer of Gloversville has also been elected to succeed the late Lucian L. Shedden.

---

Dr. H. L. Spoul of Corning has recently had an unusual interference with his practice. His office is up-stairs in a business block and workmen, repairing an adjoining building took down the only stair case, marooning the doctor in his office. He escaped by way of the roof but, for some time, his office with its equipment was inaccessible.

---

Dr. Ira W. Livermore of Gowanda, returned Feb. 10, from a business trip to Alabama.

---

Dr. C. H. Thomas of Silver Springs, has gone to Pine Bluff, N. C., for the rest of the winter.

---

Dr. Marshall Clinton of Buffalo, is taking the Panama trip.

---

Dr. Michael A. Conboy, P. & S., Baltimore, 1901, formerly located at Holyoke, Mass after devoting considerable time to post-graduate study, has opened an office at 482 Delaware Ave., Buffalo. Practice limited to eye, ear, nose and throat.

---

Dr. George D. Stilson has removed from Buffalo to Niagara Falls.

---

Dr. Willis B. Gifford of Attica, was elected President of the New York State Homeopathic Society, at the convention in Albany, in February.

---

Dr. William S. McGill, the director of the State Hygiene Treatment and Animal Experimentation on Feb. 15. This address constituted one of a series on Popular Medical Topics given by the Monroe County Medical Society.

---

Drs. Regina Flood Keyes and Mabel Flood have returned from Panama.

